# Supplementary material for: How ornithopters can perch autonomously on a branch
Source: Nat Commun. 2022 Dec 13;13:7713. doi: 10.1038/s41467-022-35356-5 (PMC9747916; doi:10.1038/s41467-022-35356-5)
Supplement: Supplementary file 1 — Supplementary Information [file 41467_2022_35356_MOESM1_ESM.pdf]

SUPPLEMENTARY INFORMATION

How ornithopters can perch autonomously on a branch

This file contains the following supplementary information:

Supplementary Method 1. Re-opening mechanism description.
Supplementary Method 2. Force/impact modeling of the leg.
Supplementary Method 3. Parametric design of the leg.
Supplementary Method 4. Mathematical expressions of the leg dynamic model.
Supplementary Method 5. Electronics description.
Supplementary Method 6. Assembly steps for the perching flapping-wing robot.
Supplementary Method 7. Power analysis.

Supplementary Table 1. Summary of key robot characteristics.
Supplementary Table 2. Optimization parameters.
Supplementary Table 3. Error values for different flight experiments and setpoints.
Supplementary Table 4. Statistical analysis of flight results.
Supplementary Table 5. List of components and source.

Supplementary Fig. 1. Re-opening system tension test.
Supplementary Fig. 2. Servo actuator comparison.
Supplementary Fig. 3. Schematic of the leg with the hip joint.
Supplementary Fig. 4. Validation of the analytical model.
Supplementary Fig. 5. Data of the active-controlled leg.
Supplementary Fig. 6. Branch detection sensor.
Supplementary Fig. 7. Control actions in flight.
Supplementary Fig. 8. Flight experiment setup.
Supplementary Fig. 9. Payload breakdown.
Supplementary Fig. 10. Launcher assembly.
Supplementary Fig. 11. Launch adapter.
Supplementary Fig. 12. 3D view of the re-opening mechanism.
Supplementary Fig. 13. Leg exploded view.
Supplementary Fig. 14. Tail assembly view.
Supplementary Fig. 15. Outdoor branch grasping.
Supplementary Fig. 16. Branch diameter parametric analysis.
Supplementary Fig. 17. Model response vs real response in typical flight.
Supplementary Fig. 18. Failed flight trajectories.
Supplementary Fig. 19. Electronics schematics.

Supplementary Method 1. Re-opening mechanism description.

We choose the smallest, widely available motor with reduction, constraining the tube size to a minimum of 13 mm inner diameter, see Supplementary Fig. 12. The entire motor-leadscrew assembly is held by an aluminum adapter, illustrated in Supplementary Fig. 12-D, that is fixed by three screws to the carbon fiber tube. Using this design, the assembly can be fully disassembled. The selected motor is a high-power micro-geared DC motor. After a $1:380$ reduction, the output of the motor drives a 3 mm leadscrew. This gives a 55 mm range to the carriage traveling on the leadscrew. Rotation between the carriage and the tube is locked by a set of parallel moon-shaped rails and friction is minimized thanks to the Polytetrafluoroethylene (PTFE) and brass composition of the carriage. This drive system operates in a pull configuration and can pull up to 200 N of force (see the tension experiment Supplementary Fig. 1). Previous push configuration experiments have revealed that under compression, the leadscrew deforms. This significantly reduces the maximum possible force. To perform an opening maneuver, the Dyneema tendons are pulled backward by the carriage, lifting the claws, as indicated by the arrows in Fig. 2-B. The tendons (in yellow) deviate via a micro ball-bearing tube insert (Supplementary Fig. 12-B). The insert slides into the leg tube and is locked in place thanks to a transverse M2 screw. This screw also serves as the shaft for the ball bearing.

Supplementary Method 2. Force/impact modeling of the leg.

With the configuration of the leg set, the parameters of the system were selected following an optimization process based on the geometric constraints and allowable stress in joints. A dynamic model of the system is developed to calculate the parameters of the mechanism that minimize the damage after the impact during the perching maneuver. The dynamic model estimates the motion of the system and the stresses in the joints for a given impact force. The "L" shape link of the system which is actuated at its base is depicted in Fig. 2-F. The impact applies an impact force F_{I} [N] to the structure. It is considered as an impulse signal and it is verified using a finite-element-based multi-body dynamics software. The value of this force depends mostly on the kinetic energy of the system before the impact and the rigidity of the branch and its frame. Then, the angular motion, which is defined by θ_1 as a function of the applied force, can be calculated by imposing the balance of torques in the first joint. The moment in the reference point is defined as the vector product of the forces and the distance from the reference point to the forces:

$$I_{\mathrm{T}}\ddot{\theta}_1 = \vec{\boldsymbol{r}}_{\mathbf{1,2}} \times \vec{\boldsymbol{F}}_{\mathbf{e}} + \vec{\boldsymbol{r}}_{\mathbf{1,3}} \times \vec{\boldsymbol{F}}_{\mathbf{i}} + \left(\sum_{i=1}^{3} m_i.\vec{\boldsymbol{r}}_{\mathbf{1},\boldsymbol{m_i}}\right) \times \vec{\boldsymbol{g}},$$

where I_{T} is the total inertia of the leg with the hip joint, $\vec{\boldsymbol{F}}_{\mathrm{e}}$ is spring force, and $\vec{\boldsymbol{g}}$ denotes gravity. The mass of the different parts of the manipulator is considered to be concentrated in their center of gravity: the mass of the links m_1, m_2 and the mass of the claw, m_3. The distance vector between the first rotational joint and the k-th joint is defined as $\vec{\boldsymbol{r}}_{\mathbf{1},\boldsymbol{k}}$ and the distance vector between the rotational joint and the concentrated mass i is defined as $\vec{\boldsymbol{r}}_{\mathbf{1},\boldsymbol{m_i}}$ (see Supplementary Fig. 3). Then, the analytical expression of the stress in the joints of the structure can be expressed as a function of the parameters of the system, the motion of the system, and the impact force:

$$\left[R_{x,1}, R_{z,1}, R_{x,2}, R_{z,2}, M_{y,2}, R_{x,3}R_{z,3}, M_{y,3}\right]^T = \mathbf{F}\left(\theta_1, \dot{\theta}_1, \ddot{\theta}_1, \Phi, F_{\mathrm{I}}\right),$$

where $R_{x,i}R_{z,i}, M_{y,i}$ are the stress in the joint i. If k_{e} and ν_{e} are the elastic constant and the damping coefficient of the spring-damper mechanism, then $\Phi = \left(m_1, m_2, m_3, l_1, l_2, k_{\mathrm{e}}, \nu_{\mathrm{e}}, c_x, c_y\right)$ are a vector including the parameters of the system. Thus, the dynamic of the system and the stress in the joints can be expressed as a function of the impact force $\vec{F}_{\mathrm{I}}$. More details about these expressions can be found in the supplementary material Supplementary Method 4. Simulations were carried out with multi-body dynamics software to validate the expressions above. The total mass of the mechanism, M, was considered 700 g, the angle of the impact 0 rad and the speed of the ornithopter before the impact, v, varied in the range 2-4 m/s. The results show that the developed model is accurate, allowing us the use of it for the optimization of the parameters of the mechanism (see Supplementary Fig. 4). The multibody dynamics software used in this work was ADAMS together with MATLAB/Simulink.

Supplementary Method 3. Parametric design of the leg.

The size and weight of the leg must be compatible with the flapping robot, which imposes a careful selection of the components. A parameter optimization within a logical range will define the best set. The objective of the parameter optimization is to minimize the weight of the leg and claw subsystem along with the applied force to the servomotor joint during the impact. The servomotor joint is the most vulnerable part of the leg and claw. The gear tooth stress within the servo is proportional to the torque at the input of the mechanism; therefore, to protect the gears, the resultant torque in the servo joint must be minimized as well. The applied force on the servo joint is $F_{\mathrm{J}}(t) = \left[R_{x,1}(t), R_{z,1}(t)\right]^T$, and the weight of the subsystem includes the mass of the first and second link and claw. Considering the force, angular momentum, and the mass of the leg, the following cost function is introduced:

$$J = \max\left(I_{\mathrm{T}}\ddot{\theta}_1(t)\right)\max\left(\sqrt{R_{x,1}^2(t) + R_{z,2}^2(t)}\right) + (m_1 + m_2 + m_3),$$

which imposes a non-convex optimization problem. The optimization parameters are selected as

$$\mathbf{x} = \{l_1, l_2, c, l_0, m_1, m_2, m_3, k_{\mathrm{e}}, \nu_{\mathrm{e}}\},$$

where the geometric parameters are presented in Fig. 2-F. The parameters, lower and upper bound of the variables are presented in Supplementary Table 2. These ranges were selected based on the possibility of the changes in geometry and physical properties of available springs and mechanical parts such as servo motors, etc. The particle swarm optimization (PSO) is used to find the best set of variables to satisfy the cost function; the PSO is an effective multi-objective optimization tool to solve constrained problems. A number of 100 particles and 1000 iterations are set for the PSO algorithm. The inertia component of PSO is set $w = 0.5$, the cognitive component $c_1 = 1.8$, the social component $c_2 = 1.5$, and the velocity damping factor $k = 0.1$. The details of PSO and interpretation of the mentioned settings are in reference textbooks. Applying the PSO, the optimal solution is found $\mathbf{x}_{\mathrm{best}}$, reported in Supplementary Table 2, with a leg mass of 0.1703 kg. Considering the available servomotor and off-the-shelf springs, the selected parameters (design parameters or final ones) are with the slightest possible deviation with respect to the optimal answer. Selecting the $\mathbf{x}_{\mathrm{final}}$, the optimal mass from the PSO simulation increased to 0.1703 kg.

Supplementary Method 4. Mathematical expressions of the leg dynamic model.

We developed a dynamic model that computes the motion of the system and the stress in the joints for a determined impact force. The system is depicted in Fig. 3-G. The mass of the different elements of the manipulator is considered lumped in its center of gravity: The mass of the first part, m_1, the mass of the second part, m_2, and the mass of the claw, m_3. The lengths of the first and second parts are l_1 and l_2, respectively. The relation between the angle φ and θ_1 is:

$$sin\,\varphi = \frac{\sqrt{c_x^2 + c_y^2}\,sin(\theta_1 - \beta)}{l_s(\theta_1)},$$

where β, c_x, and c_y are defined in Fig. 2-G, and

$$l_s(\theta_1) = \sqrt{\left(l_1\,sin\,\theta_1 - c_y\right)^2 + (l_1\,cos\,\theta_1 - c_x)^2}.$$

The force exerted on the spring-damper mechanism is defined as $F_e = k_e(l_s - l_o) + \nu_e \frac{dl_s(\theta_1)}{dt}$ where $l_s(\theta_1)$ is the length of the spring, l_o is the preload length of the spring; k_e, and ν_e are the elasticity constant and the damping coefficient of the spring-damper mechanism. Then, the equation of the motion which is defined by θ_1 as a function of the applied force can be calculated by imposing the balance of torques in the first joint:

$$\begin{aligned} I_T\ddot{\theta}_1 = -l_1\left(k_e(l_s(\theta_1) - l_o) + \nu_e \frac{dl_s(\theta_1)}{dt}\right)\frac{\sqrt{c_x^2 + c_y^2}\,sin(\theta_1 - \beta)}{l_s(\theta_1)} \\ - F_i(l_1\,sin\,\theta_1 + l_2\,sin(\theta_1 - \theta_2)) \\ + g\left(\left(\frac{m_1}{2} + m_2 + m_3\right) l_1\,cos\,\theta_1 + \left(\frac{m_2}{2} + m_3\right) l_2\,cos(\theta_1 - \theta_2)\right). \end{aligned}$$

The torque expression can be simplified as the vector product of the forces and the distance from the reference point to the force. The vector distance between the rotational joint and the k-th joint is defined as $\vec{r}_{1,k}$ and the distance vector between the rotational joint and the concentrated i-th mass is defined as $\vec{r}_{1,m_i}$ (see Supplementary Fig. 3).

$$I_T\ddot{\theta}_1 = \vec{r}_{1,2} \times \vec{F}_e + \vec{r}_{1,3} \times \vec{F}_i + \left(\sum_{i=1}^{3} m_i.\vec{r}_{1,m_i}\right) \times \vec{g},$$

where I_T is the total inertia of the mechanism and can be written as follows

$$I_T = I_{c,1} + m_1\left(\frac{l_1}{2}\right)^2 + I_{c,2} + m_2\left(l_1^2 + \left(\frac{l_2}{2}\right)^2 + l_1 l_2\,cos\,\theta_2\right) + m_c(l_1^2 + l_2^2 + 2l_1 l_2\,cos\,\theta_2),$$

where $I_{c,1}$ and $I_{c,2}$ are the inertia of the first and second parts of the "L" shaped link with respect to its base. Once the equation of motion is obtained, we can calculate the analytical expression of the stress in the joints, considering the free-body diagram in Supplementary Fig. 3. Notice that the

bending stress of the first joint is zero because the system can rotate freely around this point. The acceleration of the center of mass of the two links and the claw are defined as follows:

$$\ddot{\vec{P}}_1 = \begin{bmatrix} \ddot{x}_1 \\ \ddot{z}_1 \end{bmatrix} = \begin{bmatrix} -\frac{l_1}{2}\dot{\theta}_1^2 \cos\theta_1 - \frac{l_1}{2}\ddot{\theta}_1 \sin\theta_1 \\ -\frac{l_1}{2}\dot{\theta}_1^2 \sin\theta_1 - \frac{l_1}{2}\ddot{\theta}_1 \cos\theta_1 \end{bmatrix},$$

$$\ddot{\vec{P}}_2 = \begin{bmatrix} \ddot{x}_2 \\ \ddot{z}_2 \end{bmatrix} = \begin{bmatrix} -\dot{\theta}_1^2\left(l_1 \cos\theta_1 + \frac{l_2}{2}\cos(\theta_1-\theta_2)\right) - \ddot{\theta}_1\left(l_1 \sin\theta_1 + \frac{l_2}{2}\sin(\theta_1-\theta_2)\right) \\ \dot{\theta}_1^2\left(l_1 \sin\theta_1 + \frac{l_2}{2}\sin(\theta_1-\theta_2)\right) - \ddot{\theta}_1\left(l_1 \cos\theta_1 + \frac{l_2}{2}\cos(\theta_1-\theta_2)\right) \end{bmatrix},$$

$$\ddot{\vec{P}}_3 = \begin{bmatrix} \ddot{x}_3 \\ \ddot{z}_3 \end{bmatrix} = \begin{bmatrix} -\dot{\theta}_1^2(l_1 \cos\theta_1 + l_2 \cos(\theta_1-\theta_2)) - \ddot{\theta}_1(l_1 \sin\theta_1 + l_2 \sin(\theta_1-\theta_2)) \\ \dot{\theta}_1^2(l_1 \sin\theta_1 + l_2 \sin(\theta_1-\theta_2)) - \ddot{\theta}_1(l_1 \cos\theta_1 + l_2 \cos(\theta_1-\theta_2)) \end{bmatrix}.$$

Then the following expressions represent the stress in the joints:

$$R_{x,1} = -F_i - F_e \cos\varphi_2 + m_1\ddot{x}_1 + m_2\ddot{x}_2 + m_3\ddot{x}_3,$$
$$R_{z,1} = -F_e \sin\varphi_2 + m_1\ddot{z}_1 + m_2\ddot{z}_2 + m_3\ddot{z}_3 + g(m_1 + m_2 + m_3),$$
$$R_{x,2} = -F_i + m_2\ddot{x}_2 + m_3\ddot{x}_3, \qquad R_{z,2} = m_2\ddot{z}_2 + m_3\ddot{z}_3 + g(m_2 + m_3),$$
$$M_{y,2} = l_2 \cos(\pi - \theta_1 - \theta_2)\left(\frac{m_2\ddot{z}_2}{2} + m_3\ddot{z}_3 + \frac{m_2 g}{2} + m_3 g\right)$$
$$+ l_2 \sin(\pi - \theta_1 - \theta_2)\left(\frac{m_2\ddot{x}_2}{2} + m_3\ddot{x}_3\right),$$
$$R_{x,3} = -F_i + m_3\ddot{x}_3, \qquad R_{z,3} = m_3\ddot{z}_3 + m_3 g, \qquad M_{y,3} = 0.$$

The stress in the joints can be defined as a function of the impact force, the equation of motion, and the parameters of the system as follows:

$$\left[R_{x,1}, R_{z,1}, R_{x,2}, R_{z,2}, M_{y,2}, R_{x,3}R_{z,3}, M_{y,3}\right]^T = \mathbf{F}\left(\theta_1, \dot{\theta}_1, \ddot{\theta}_1, \Phi, F_i\right),$$

where $\Phi = \{m_1, m_2, m_3, l_1, l_2, k_e, v_e, c_x, c_y\}$ is the vector parameter of the system. The validation of stress in the first joint is obtained by using the analytical model presented in this work and the obtained results using ADAMS together with MATLAB/Simulink. The graphics in Supplementary Fig. 4 show the perfect match between the model and the simulation. The stress in the other joints is also validated. For those simulations, the total mass of the mechanism, M, was considered $700(\mathrm{g})$, the angle of the impact 0° and the speeds of the ornithopter before the impact, v, were 2,3 and $4\left(\frac{\mathrm{m}}{\mathrm{s}}\right)$. The results show the high accuracy of the developed model, obtained for the optimization of the parameters. The impact force is similar to an impulse signal of $20(\mathrm{ms})$ whose magnitude increases proportionally to the speed. Moreover, Supplementary Fig. 4 shows the components of the stresses at the first joint (where the motor is located) and the equivalent reaction torque at this joint. In the continuous (red) line, the results of the software are depicted, whereas in the dashed (blue) line the results using the analytical model are plotted.

Supplementary Method 5. Electronics description.

The electronics on-board the perching flapping-wing robot is based on a central, unifying PCB. See schematic in Supplementary Fig. 19. This printed circuit board carries all the low-level components and sensors while also linking with the external peripherals. The peripherals all connect via JST-GH micro-connectors. This enables full modularity, separation of components, and simpler debugging. Overall, the flight companion computer, flapping actuation, tail, leg-claw system can all be swapped in a matter of seconds. This fully modular approach has been highly beneficial in the context of experiment-intensive flight tasks.

Microcontroller. The microcontroller used on-board the perching robot is a STM32L432KBUx. This microcontroller is selected for its small format, number of GPIOs, and mostly its availability (during the chip shortage of 2021). It is connected to an external clock and is programmed via an ST-Link connector, located towards the rear of the body.

Flapping Actuation. The flapping wing motion is generated by a $150(\mathrm{W})$ brushless motor from Hacker. An ESC powers the motor with up to $35(\mathrm{A})$, far above the required maximum current of the motor. As with most ESCs, control of the brushless motor happens through a pulse width modulation (PWM) line. The microcontroller, therefore, sends a command corresponding to the power output, i.e. not the frequency. With a total weight of $700(\mathrm{g})$, a throttle of 65% is required for level flight. Climbing is achieved in the perching experiments at throttles of up to 90%.

Power. The power is supplied by a 4S battery with a capacity of $450(\mathrm{mAh})$. This battery is small, as the application here does not require long flight times. The battery voltage is then regulated to three different levels. The regulators chosen have a 95% efficiency, state-of-the-art small footprint, $5.5 \times 5.5(\mathrm{mm})$, and $3(\mathrm{A})$ handling capacity. In addition, only two external resistors are required to select the output voltage. The three levels are:

1. The first regulators power a $5(\mathrm{V})$ bus to a Nanopi Neo Air and an optional Event Camera and KHADAS VIM3.
2. The second regulator uses low-voltage electronics components on the PCB (microcontroller, ESC, RC-receiver, and the sensors).
3. The last regulator is used for the servo actuators, which can function between $5(\mathrm{V})$ and $10(\mathrm{V})$, depending on servo requirements.

Flight components.

4. RC Receiver. The robot connects via an RC Futaba receiver with remote control. The microcontroller is programmed with an override switch so that manual control can always be regained if the autonomous flight is not working properly.
5. Tracking IR Led. Good indoor tracking of the robot is possible thanks to the onboard IR LEDs that yield better detection than reflective balls. The robots feature a total of six LEDs, located asymmetrically to avoid orientation errors. The LEDs are connected in series with a 100Ω resistor, limiting the current to $70(\mathrm{mA})$, experimentally determined to give the best detection within the Optitrack system. The two center-located LEDs stick out through the wing membrane, as apparent in the PCB shape. The proximity of those two points is slightly too close and should be moved at least $8(\mathrm{cm})$ apart.

6. GPS/IMU. The PCB carries the required connector (located after the flapping hinge) to carry a VectorNAVVN200 sensor. This high-end sensor is connected to the companion computer to feed position and attitude information when indoor tracking is not available. The sensor connects horizontally and is located close to the center of mass.

Additional Sensors. Several sensors are present on the PCB, as listed here:

7. Magnetic encoder. The DRV5055 sensor is a ratiometric linear hall effect sensor. This sensor was selected for its simplicity as it only needs one external component. A magnetic ring is placed on the intermediate gear shaft, and this sensor will be close to it. As the ring turns, the magnetic field will be seen oscillating by the sensor. The sensor analog output is connected to the microcontroller. The measurements can be used to know the velocity of the flapping motor, giving 12 points per turn (thanks to the gear reduction).
8. Current sensor. We select an ACHS-7122-500E sensor. It is a Half Effect-based isolated linear current sensor, which has a $\pm 20(\mathrm{A})$ functional range.
9. Voltage sensor. A resistive bridge with a Zener diode to protect the microcontroller input is used to measure the battery voltage. Power consumption can therefore be obtained, together with the current measure.
10. Wind sensor. Two SDP37 are visible on the head of the flapping-wing robot. Both sensor tubes are planned to have an angle offset in their orientation, which can be used to calculate the wind velocity and pitch incidence.
11. Ultrasound. This external distance sensor permits readings of the altitude to the ground. The sensor can detect the ground at up to $6.45(\mathrm{m})$ with a $2.5(\mathrm{cm})$ resolution. The ultrasound sensor sends the measure to the microcontroller via an analog signal.

Supplementary Method 6. Assembly steps for the perching flapping-wing robot

The assembly steps to reproduce a perching flapping-wing robot are presented here, based on the list of components in Supplementary Method 4.

1. **Body plate.** Cut the profile of the left side of the body in a 1.5 mm woven carbon fiber plate. Install the 20 mm spacers at each corner location on the cut plate. Install both 4 mm and 6 mm inner diameter bearings.

2. **Transmission.** The axis is lathed from stainless steel rods to size, with a slight end chamfer for precision. 0.5mm diameter grooves are cut to secure circlips, keeping the axis in place axially. On the largest gear assembly, the axis is drilled and threaded diametrically at both extremities and the gear location, with the same orientation. A 3D-printed guide that holds the axis helps to drill the holes on a vertical manual drill. Insert both gears on both axes, which should have a slight compression fit. The largest gear's rotation with the axis is locked by the M3 screw which should fit into the threaded hole made earlier. Slide the 2 larger gear-axis assemblies into the bearings in the carbon fiber plate. Fit the brushless motor with the small gear, and secure it with the grub screw. The motor can now be fixed in the assembly. For that slide 2 M3 screws into the holes for this purpose in the carbon plate. Insert 2x 10 mm spacers on those screws. Next, the motor can be inserted through the large hole in the PCB and secured on top of the screws.

3. **PCB.** Insert the 4 mm and 6 mm inner diameter into the fully populated PCB. At this point, the PCB can slide onto the two axes. With 4x M2, screw the PCB to the spacers.

4. **Tail fitting.** The 3D-printed tail compression pieces (4x) can be slid between PCB and carbon plate, aligned with the tail holes. Insert 4x M2x25 screws as well as the carbon fiber tail tube (50 cm long). Secure the tail through compression with 4x M2 nuts at the end of the screws. Pass the tail cables through the tube.

5. **Wing-roots.** Prepare the aluminium wing roots by M2 threading half of the compression fit holes, the other half is a through-hole. Insert 3x6 mm bearings into the two extremities of the wing roots. The wing-roots-axis assembly can be assembled now. Separately, insert the 3 mm flapping axle into the 3D-printed Back flapping axis holders. Slowly pushing the axle through that part, insert both wing-roots, with a washer between wing-roots and 3D holders. Then insert the Middle flapping axis holder and the second side of the wing-roots. The assembly is complete with the final Front flapping axis holder. The order of the parts on the axle should be: H-w-Y-w-Y-w-H-w-Y-w-Y-w-H, where H=Holder, w=washer, Y=Y extremity of the wing roots. The flapping axis assembly can now be inserted into the body assembly. It is fixed in the top section of the PCB and carbon plate with 3x M3x25 screws and nuts. Tightly screw down.

6. **Discrete Electronics.** The discrete electronics components can be added. The Nanopi Air slides in between the body plate and is secured in place by its double header connector row. The RC transceiver is connected and secured to the carbon body plate with a small ziptie. The same goes with the ESC, on the PCB side.

At this point the body assembly is complete. Wings, tail, and leg can be added in a modular manner.

1. **Wing assembly.** The 6/4 mm OD/ID carbon fiber woven tubes are cut to the correct length as well as the 1.5 mm diameter 5 chord rods. The wing skeleton consists of a perpendicular arrangement of chord rods to the main spar, held in place by 3D-printed brackets. Using hot glue (in addition to the slight compression fit) to secure the brackets has proven sufficiently strong yet allows for simple repair of the chord rods, should one break on impacts. The ripstop nylon membrane needs to be cut to the approximate shape of the full double wings with a 5 cm margin. It can then be tensioned (by hand) and glued to the top side of the afore-mentioned 3D brackets with a drop of superglue. The chord rods are fixed to the membrane with the help of strips of ripstop tape. Repeat with the other wing. The pair of wings can now be inserted into the robot, in a high dihedral position.

2. **Tail assembly.** See the assembly in Supplementary Fig. 14. Start by assembling the elevator. Using dyneema or Kevlar thread, tie the 3 2-mm carbon elevator rods between the 2 carbon fiber plates of the elevator root. Cover the threads with epoxy. Insert the pivots (3D-printed parts) between the 2 plates, bond with epoxy. To finish the elevator tail skeleton, slide the 3 printed fittings on each rod that will hold the transverse rods which keep the tension in the fabric. Bond in place, insert the cut transverse rods. Cover the elevator skeleton with ripstop nylon, using superglue on the sides (membrane to rods) and a ripstop tape cover on the central rod. Screw the rudder servo down with 2x M2 screws. The rudder can be assembled in a similar manner. Insert the rods into the rudder root (3D-printed bracket). The pivoting point of the rudder root is a 30mm M3 screw which is secured in the elevator double-plate. Attach a horn to the rudder servo and link to the rudder with a Z-shaped metal wire (1 mm).
   Build the sandwich structure that will hold the elevator-rudder assembly. The top and bottom plates are cut in 1.5 mm carbon fiber plates. Using 6x M2x20, link together the sandwich structure as follows: C-P-T/S/B-P-C. Where C=Carbon plate, P=Printed 3D compression holder, T=Tail tube, S= Servo, B=Bearing. Thanks to this method, the servo, bearing, and tail tube are compression held. Insert the sandwich structure at the end of the tail tube, making sure not to pinch the cables. Install the IR LEDs on the top part of the assembly. Add the elevator servo horn. Fix the elevator-rudder assembly to the horn on one side. Add the second rotation point with an M3 screw that fits in the bearing.

3. **Leg assembly.** The leg is composed of an active hip servo joint and the leg tube. See the servo assembly in Supplementary Fig. 13. The leg design is modular, and it can be assembled independently from the robot or the claw. Secure the leg servo between the 2 carbon plates with M2x25 screws on the bottom part. The 2 M3x30 screws on the top

portion also hold the back rotation carbon plate. This plate, which is flush with the backside of the servo, is responsible for holding the axis (screw) and ball bearing. Insert the top carbon plates which are fixed on one side of the servo horn, on the other side of the back pivot. Secure together via 2 long M3 screws and the 3D-printed compression part. This is the attachment point to the robot's tail tube. Finally, insert the leg tube on the bottom compression fit.

4. **Claw reopening.** Inside the leg tube is located the claw-reopening mechanism, see Supplementary Fig. 12. Insertion of the mechanism within the tube needs to follow the steps described here due to the lack of visibility. First, visibility can be improved via a set of 7 mm holes through the tube walls at the slider location. Then, drill 3x 3mm holes through the carbon walls at 120°, and a 3D-printed guide can help. Mount the motor, with a thrust bearing, locking the nut onto the aluminum holder (in Supplementary Fig. 12-D). Attach the dyneema tendons to the slider and screw at the end of the lead screw. Leave the tendon long (50 cm). Insert the motor assembly into the tube, secured with 3x M3x6 in the tube. The tendons should hang out on the claw side. Now, insert the tendon diverter (Supplementary Fig. 12-B), with the tendons passing through the diverter and around the ball bearings. Tendons should still hang out on the claw side. Slowly insert the diverter with the guide rails so that the rails slide alongside the carriage. Once in place, insert the M2x20 axis which goes through the bearings. The components are now all in place. Using tweezers, the tendons can be pulled out through the hole in the tube walls and attached to the claw.

5. **Claw assembly.** Claw assembly happens in three steps. First, mount the side carbon plates to the leg tube using the 3D printed compression parts and 2x M3x30. Secondly, assemble the top pair of claws and the bottom pair of claws with the 2 20 mm spacers. Mount onto the carbon side plates with 4 M3x8 mm screws, which are the pivoting points of the claws. The spring can be installed, in the closed position, with two large zip ties. The use of zip ties permits fast release of the spring force should disassembly be needed. Close the zip ties so that in the open position, the spring is extended to the maximum specified in the datasheet. Finally, the re-opening tendons can be attached to the claws, through a transverse hole in the backspacer. Add the vision linescan sensor on the same screws holding the claw onto the leg tube. The sensor can be offset back slightly to prevent damage in case of impacts into a net (see Supplementary Fig. 6-C). Adjust focus. Spikes, pads and ecoflex membrane were installed last.

Supplementary Method 7. Power Analysis

The robots' power consumption in flight strongly depends on the flapping frequency as shown in the table below. Extrapolating from in-flight measures with a curve fitting of $P = 2.66f^{2.27}$ gives a power consumption of 61.8W at 4Hz flapping frequency. This frequency has been observed experimentally in the perching level flight sections.
Power measurements collected onboard during active free flight. Note: the robot's mass in this test was 670g, or 4% lighter than the robot used in the perching demonstration.

	Frequency	Current	Power
Measured	2.9 Hz	1.8 A	29.7 W
	3.5 Hz	2.8 A	46.2 W
	4.7 Hz	5.2 A	85.8 W
	5 Hz	6.4 A	105.6 W
Extrapolated	4 Hz		61.8 W

From the total power measurements at the battery level, we need to subtract the constant, flight independent, power consumption of the onboard avionics. A breakdown of the power draw of individual subsystems is presented in the table below.

System	Power measurements
PCB	0.81 W
Nanopi companion computer	0.69 W
Current sense	0.18 W
RC receiver	0.41 W
ESC (brushless motor driver)	0.41 W
Optitrack infrared LED	0.99 W
Tail servos (rudder/elevator)	1.57-5.12 W
Avionics total	6.8 ± 1.8 W

Taking the propulsive power of the 700g flapping vehicle in level flight as 61.8 W - 6.8 W = 55W, we can scale this propulsive power for a lower weight as follow.
The power P in cruising conditions is a product of the thrust T and the cruise velocity V , that is, P = TV = DV, where the thrust is balanced by the drag D, and the lift is balanced by the weight. For a given lift-to-drag ratio, the thrust is proportional to the weight. In cruising conditions, $V \propto W^{\frac{1}{6}}$, and therefore $P \propto W^{\frac{7}{6}}$ according to Tianshu Liu et al, 2006. According to these scaling estimates, the resulting propulsive power required for a without leg and claw (which would weigh 516g) would be 39 W, or a saving of 16 W. This is equivalent to a 29% power cost for the additional perching appendage. Given a 4 second flight maneuver, the required energy expenditure is summed up in the table below.

Scenario	Duration	Energy estimate	Battery usage
Perching flight	4 s	248 J	0.93 %
Claw loading	20 s	60 J	0.22 %
Perched (servos off)	1 h	7550 J	28.00 %
Flight without claw-leg	4 s	182 J	0.68 %
Battery capacity		26700 J	100.00 %

Supplementary Table 1. Summary of key robot characteristics.

P-Flap main specifications.

Parameter	Value	Unit	Description
m	0.7	kg	Mass (w/ battery, leg, claw)
S	0.438	m^2	Wing surface
b	1.5	m	Wingspan
c	0.317	m	Mean chord
S_t	0.099	m^2	Tail surface
b_t	0.46	m	Tail wingspan
Φ	$30 - 50$	°	Flapping amplitude
D	5	°	Mean dihedral angle
f	5.5	Hz	Maximum flapping frequency
k_v	1130	rpm/V	Brushless motor K_v constant
n	42	−	Gearbox reduction
V	16.5	V	Battery voltage
C	450	mAh	Battery capacity

Supplementary Table 2. Optimization parameters.

Parameters, lower and upper bound of optimization, the best and design parameters.

	l_1	l_2	c	l_0	m_1	m_2	m_3	k_e	v_e
Unit	m	m	m	m	kg	kg	kg	$\frac{N}{m}$	$\frac{Ns}{m}$
Range low	0.04	0.12	0.06	0.06	0.065	0.025	0.045	270	0.075
Range high	0.06	0.19	0.09	0.1	0.12	0.04	0.075	450	0.125
Optimal (best)	0.04	0.19	0.06	0.1	0.065	0.04	0.0653	270	0.0857
Design (final)	0.045	0.15	0.07	0.0761	0.0832	0.0298	0.06	362.2	0.1

Supplementary Table 3. Error values for different flight experiments and setpoints.

Error in cm	2 m setpoint	1.75 m setpoint	2.25 m setpoint
1st flight error	12.86	0.63	6.72
2nd flight error	16.28	13.67	7.90
3rd flight error	19.32	5.76	7.04
Mean	16.16	6.69	7.22

Supplementary Table 4. Statistical analysis of flight results.

Analysis of successful flights:

	Vx [m/s]	Yaw [deg]	Pitch [deg]	Y [m]	Z [m]
Soft Branch 1	2.43	-8.3	23.6	-0.05	2.06
Soft Branch 2	2.07	-4.6	25.1	-0.23	2
Branch 1	2.52	1.9	26	-0.02	1.96
Branch 2	2.54	3.1	31.8	-0.07	1.95
Branch 3	2.34	4	25.8	-0.15	2.04
2nd Robot	2.8	0.52	30.3	0.02	1.99
Mean	2.45	-0.56	27.1	-0.08	2
Standard deviation	0.24	4.85	3.21	0.09	0.04
Reference value	2.5	0	30	0	2
Worst value	2.07	-8.3	23.6	-0.23	2.06

Analysis of failed flights:

	Vx [m/s]	Yaw [deg]	Pitch [deg]	Y [m]	Z [m]
Branch fail I	2.38	0.473	28.22	-0.523	1.992
Branch Fail II	2.22	1.67	26.89	-0.518	2.06
Branch Fail III	2.45	-0.1	28.23	-0.231	2.09

The trajectories from the failed flights are shown in Supplementary Fig. 18.

Supplementary Table 5. List of components and source.

Body		
Body frame	CNC-cut 1.5mm woven CF	Clipcarbono
Front shock absorber	3D printed TPU	RS
Big gear	CNC-cut aluminum/steel pinion	CATEC
Medium gear	CNC-cut aluminum/steel pinion	CATEC
Small gear	Steel	RS
Big gear axle	6mm steel	RS
Medium gear axle	4mm steel	RS
Crank shaft	CNC-cut aluminum	CATEC
Pushrods ball joints	M3 aluminum	Vueloverde
Pushrods bars	M3 treaded steel	RS
Body spacers	20mm M2/M3	Smartshapes
Flapping axle	M3 steel	RS
Flapping axle holder	3D printed PLA	RS
Screws, nuts, bearings	Stainless steel	RS

Wings

Wing roots	3D Sintered Aluminum	CATEC
Main spar	6/4mm woven tube	Clipcarbono
Chord rods	1.5mm protruded CF	Clipcarbono
Membrane	Mirai 48 Ripstop Nylon	Wind-r
Tape	Nylon Ripstop	Wind-r
Trailing edge holder	3D printed PLA	RS
Launcher connector	12/10mm woven CF tube	Clipcarbono

Tail

Structure rods	2mm protruded CF	Clipcarbono
Membrane	Mirai 48 Ripstop Nylon	Wind-r
Servos	MICRO SERVO HV BMS-M15H	Intermodel

Electronics

Full body PCB	1.6mm 4L FR4, manufactured by	PCBWay
SMD components	See Supplementary Method 3	Digikey/Mouser
Battery	Tattu LiPo battery 450mAh 4S1P	Vueloverde
Nanopi	Neo Air	Friendlyelec
Brushless motor	A20 26M 1130kV	Hacker motor
ESC speed controller	TMotor F35A	RCInnovations
RC Transceiver	Futaba r6203sbe	RCInnovations

Claw

Plates	CNC-cut 1.5mm woven CF	Clipcarbono
Tube-claw holder	3D printed PLA	RS
Spacers	20mm aluminum	Smartshapes
Spring	751-944	RS
Motor with reduction	HP 6V, 1:380, 3x55mm screw	Amazon
Thrust bearing	7806K53	123Rodamientos
Slider	Teflon/Brass	RS
Motor holder	Aluminum	RS
Tendon diverter	M2x20 + Bearing 6x2x3	RS
Tendons (fishing line)	26Kg strength Dyneema	Decathlon
Linescan sensor	Parallax TSL1401	Mouser

Legs

Tube	15/13 mm woven CF	Clipcarbono
Servo Blue Bird	BMS 922+	Intermodel
Microcontroller Seeduino	Xiao M0	Mouser
DC motor controller	TB6612	Mouser
Compression fittings	3D printed PLA	RS
Frame	CNC-cut 1.5mm woven CF	Clipcarbono

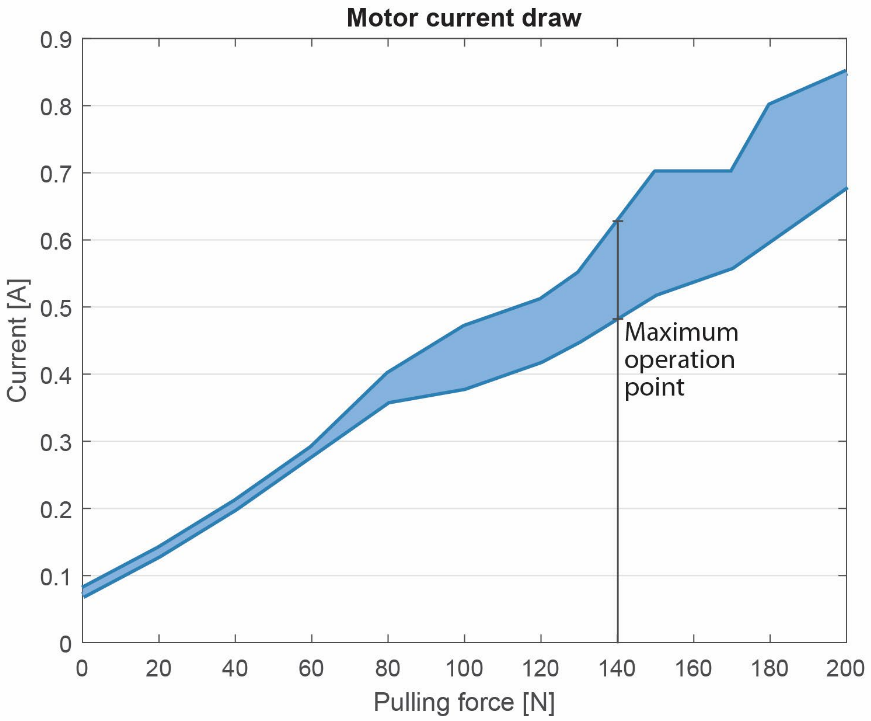

Supplementary Fig. 1. Re-opening system tension test.
Current draw of the re-opening subsystem in the leg. The 6 V geared motor drives a leadscrew slider. The current is measured with a constant load attached to the re-opening system. As the claws are opened, the tension required increases to a maximum of 140 N. At this point, the motor draw between 0.5 and 0.6 N, well below the 1.5 A limit. The subsystem is tested to load up to 200 N, giving a safety margin if friction increases due to wear. These measures are performed experimentally by suspending a variable weight load to the output tendons.

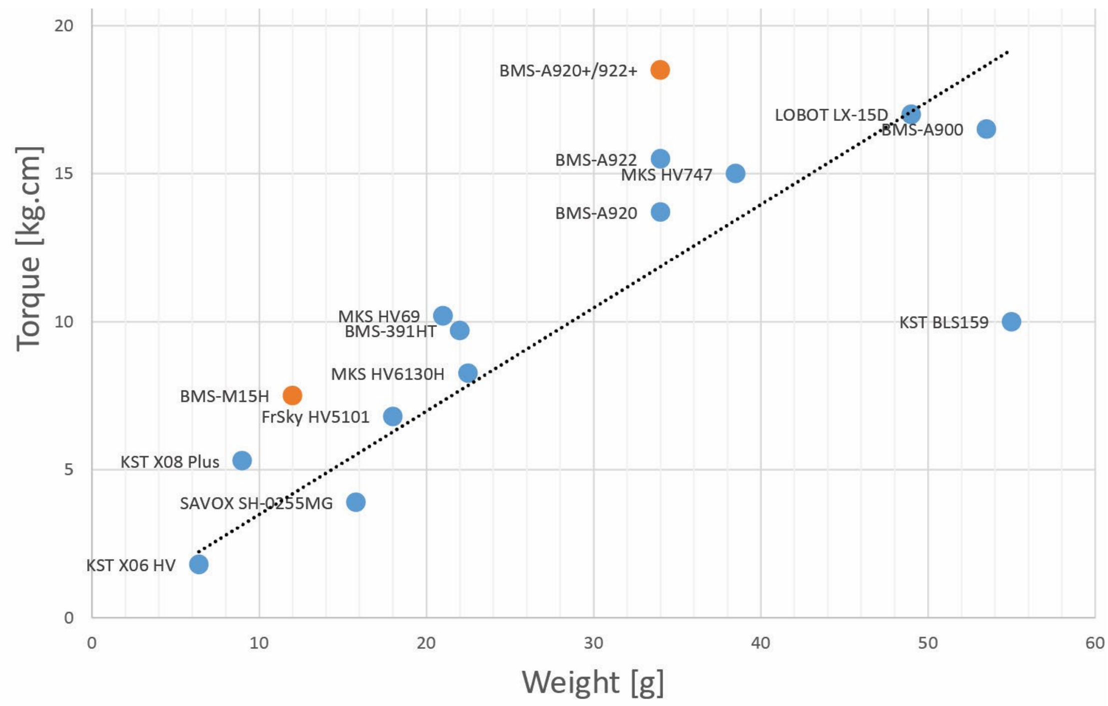

Supplementary Fig. 2. Servo actuator comparison.
A comparison between available servomotors in the market for optimizing torque/weight ratio.

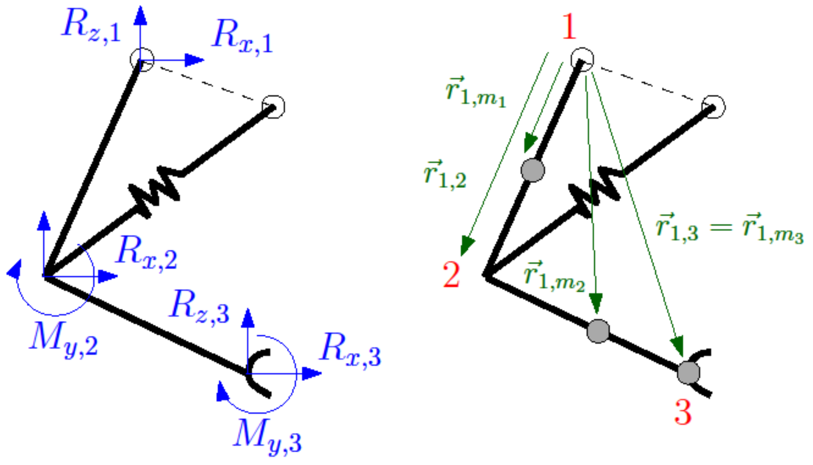

Supplementary Fig. 3. Schematic of the leg with the hip joint.
These figures represent a simplified scheme of the L shape link of the system which is actuated at its base. The variables $R_{x,i}, R_{z,i}, M_{y,i}$ are the stresses in the joint i being i = 1,..,3. The vector distance between the rotational joint 1 and the joint k is defined as $\boldsymbol{r_{1,k}(r)}$ and the vector distance between the rotational joint and the concentrated mass i is defined as $\boldsymbol{r_{1,m_i}(r)}$.

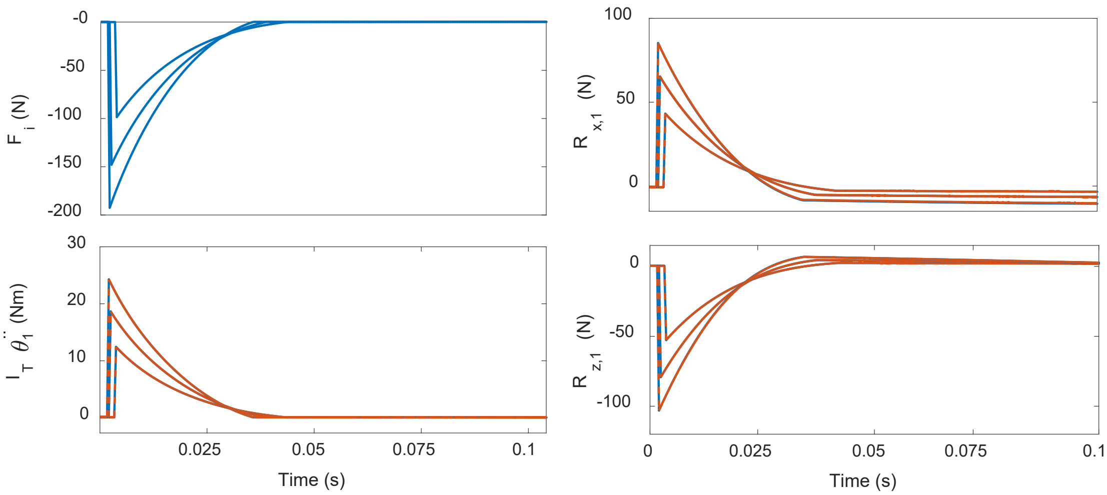

Supplementary Fig. 4. Validation of the analytical model.
Validation of the analytical model using ADAMS together with MATLAB/Simulink.

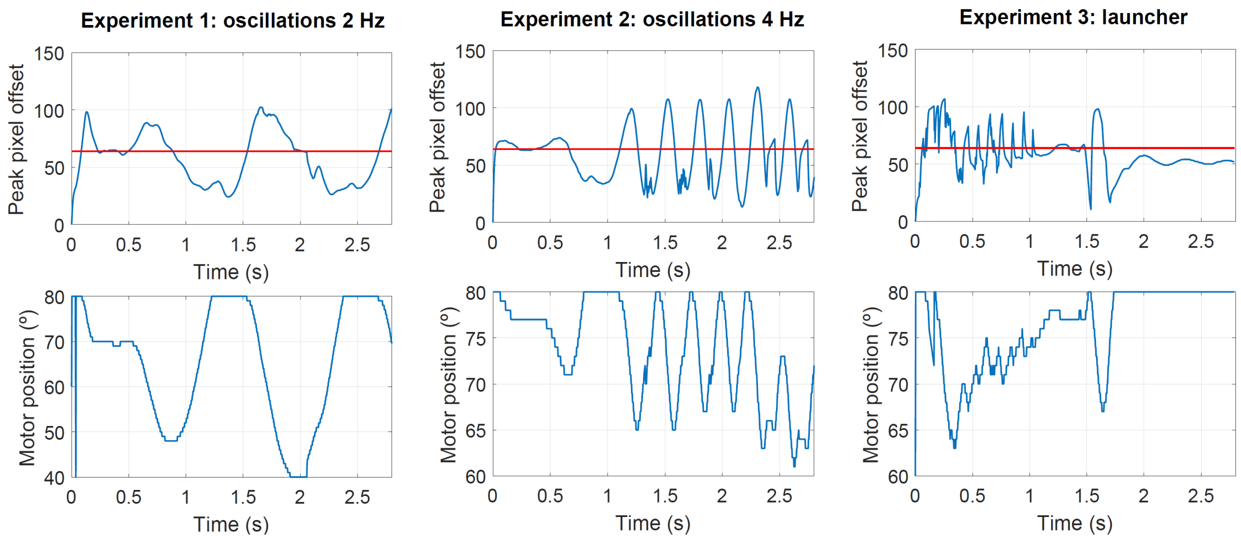

Supplementary Fig. 5. Data of the active-controlled leg.
Active leg control verification. The upper figures show the peak pixel offset (blue line) collected by the sensor and the reference value (red line). This value means that the claw is completely aligned with the branch. The lower figures show the motor position of the active leg.

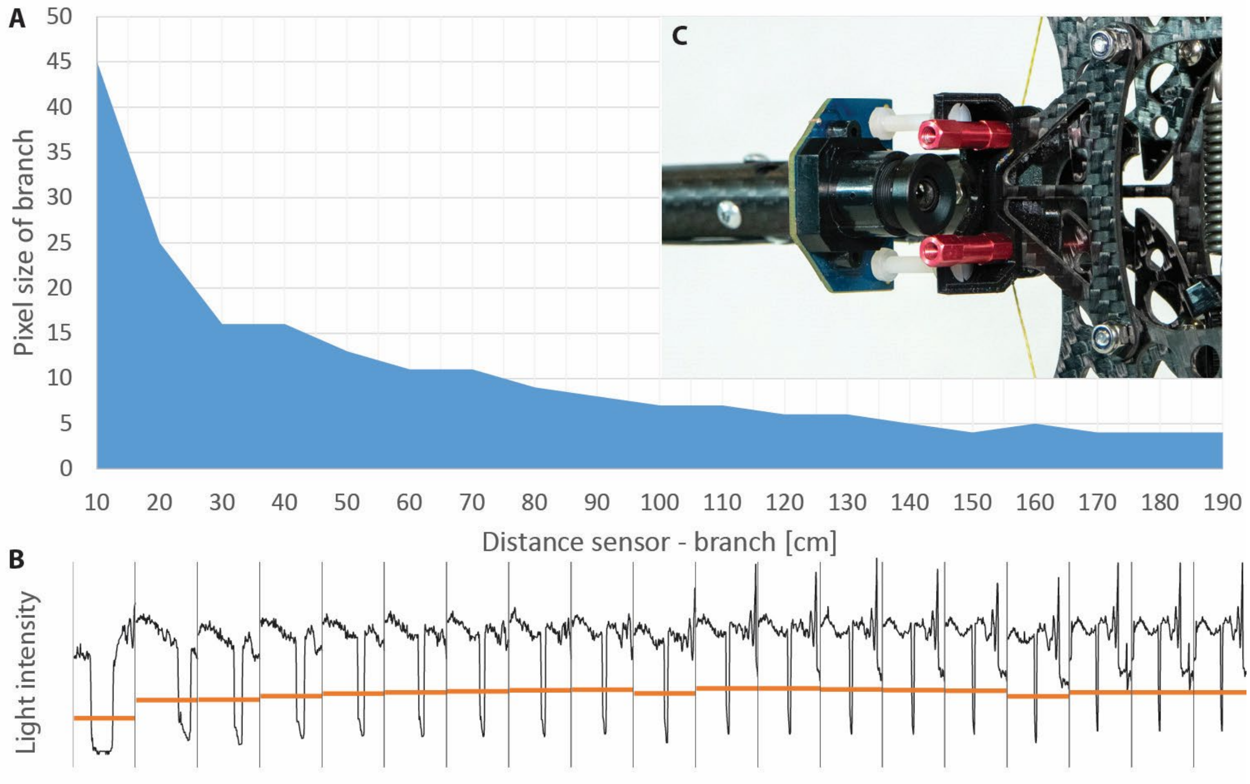

Supplementary Fig. 6. Branch detection sensor.

A. Size of the branch as a function of distance branch-sensor. The branch is detected at up to 2 m away. The lens focus is adjusted to 2 m, leading to a soft signal when close to the branch. This is visible in the softer edges of the sensor signal. **B**. Raw sensor output with increasing distance from the branch (black line), *X* axis is identical to Supplementary Fig. 6-A. Dynamic threshold calculated from the average light intensity (orange lines). The middle point of the pixels below the threshold is selected as the branch location. This is then used for the control feedback loop. **C**. Sensor mounting with the vibration absorbing nylon screws and the red 20 mm extended nuts that protect the sensor against impact with the safety net.

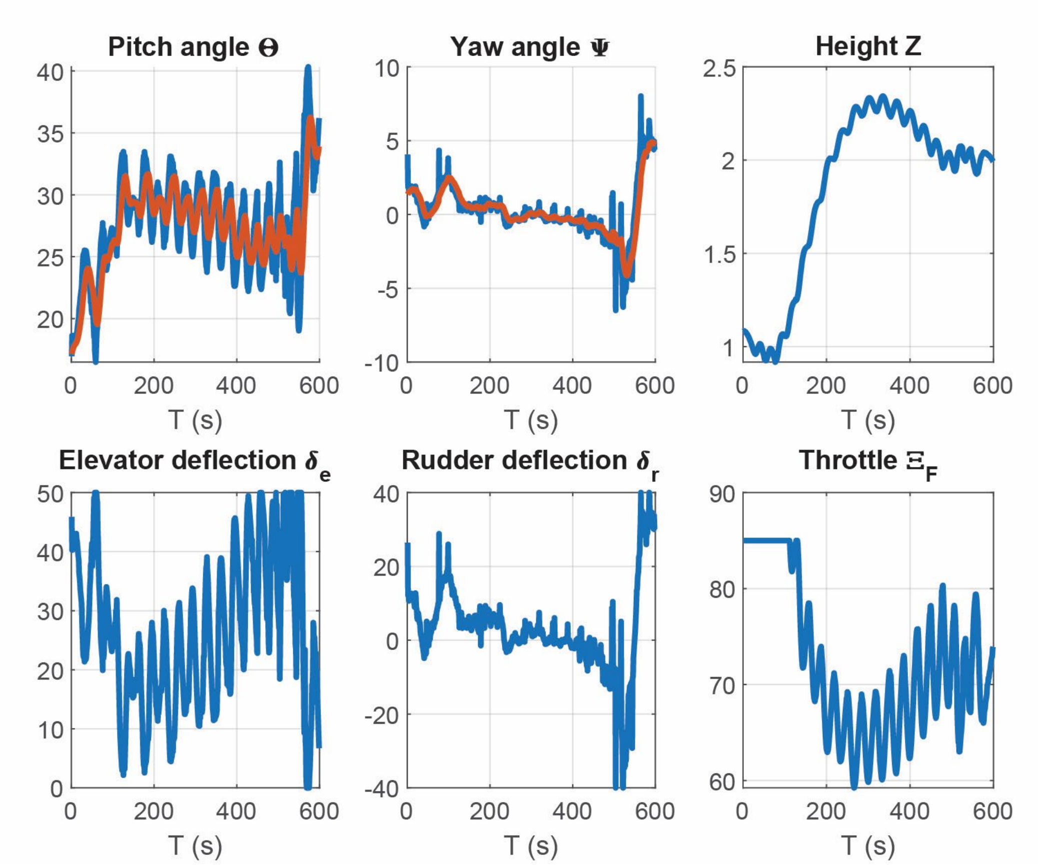

Supplementary Fig. 7. Control actions in flight.

Control data of a typical flight experiment. Three system states are received from the motion capture system: Pitch, Yaw, and Height. The corresponding flight control actions are shown in the 2nd row of graphs. The tail deflections are bounded to the physical limits of the systems, some saturation is visible. The throttle is equally bounded between 50% and 85%, keeping the wings flapping at all times but also avoiding unnecessarily large flapping frequencies which prematurely wear out the vehicles. Note: $T(s)$ represent the time in samples, captured at 120 Hz.

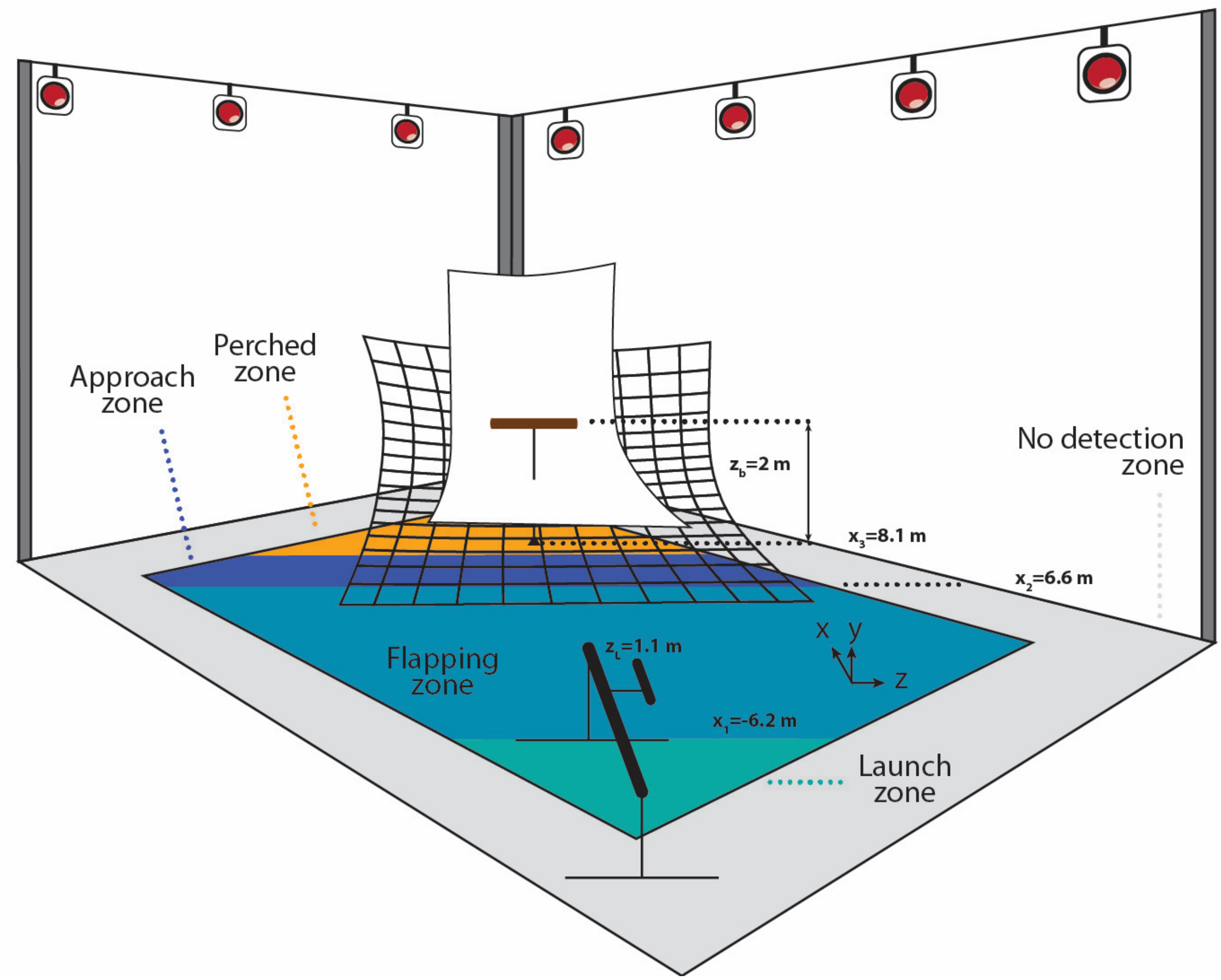

Supplementary Fig. 8. Flight experiment setup.
Representation of the test setup used for flying and perching. The robot starts in the launch zone, in a corner of the detection space. The different zones are shown, which change the robot's behavior in flight. The landing is protected with a net. A white background is placed behind the branch to improve the line-scan detection.

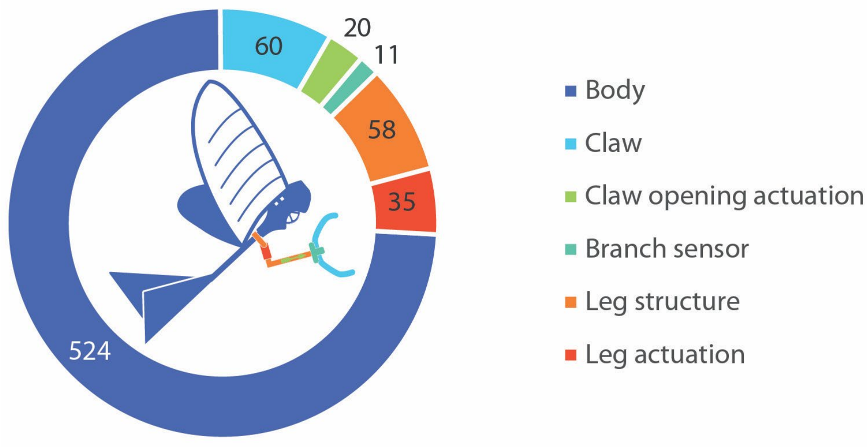

Supplementary Fig. 9. Payload breakdown.
Mass budget of the flapping-wing robot. The 524 g body consists of a fully flight-capable robot with a tail and electronics (see Ref. *(15)* for further breakdown). The rest of the indicated mass distribution shows the penalty of the additional perching system. Overall, the grasping mechanism with re-opening electronics weighs a total of 80 g, and the stabilizing leg system weighs a total of 104 g.

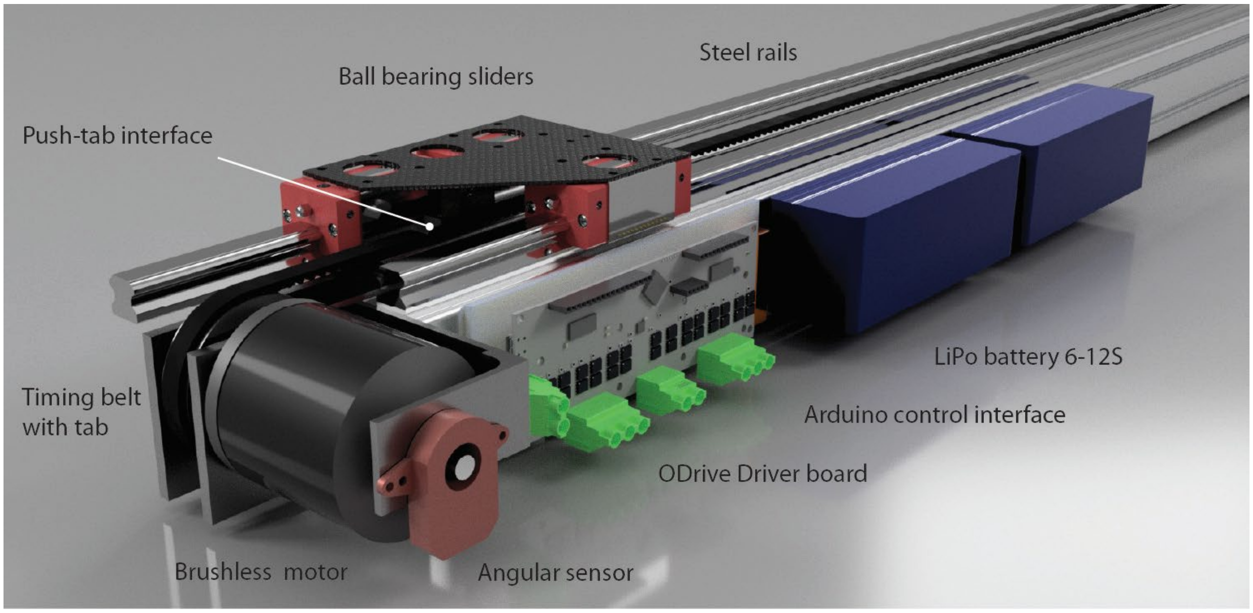

Supplementary Fig. 10. Launcher assembly.
3D representation of the launching apparatus showing the brushless motor, driver, and rails.

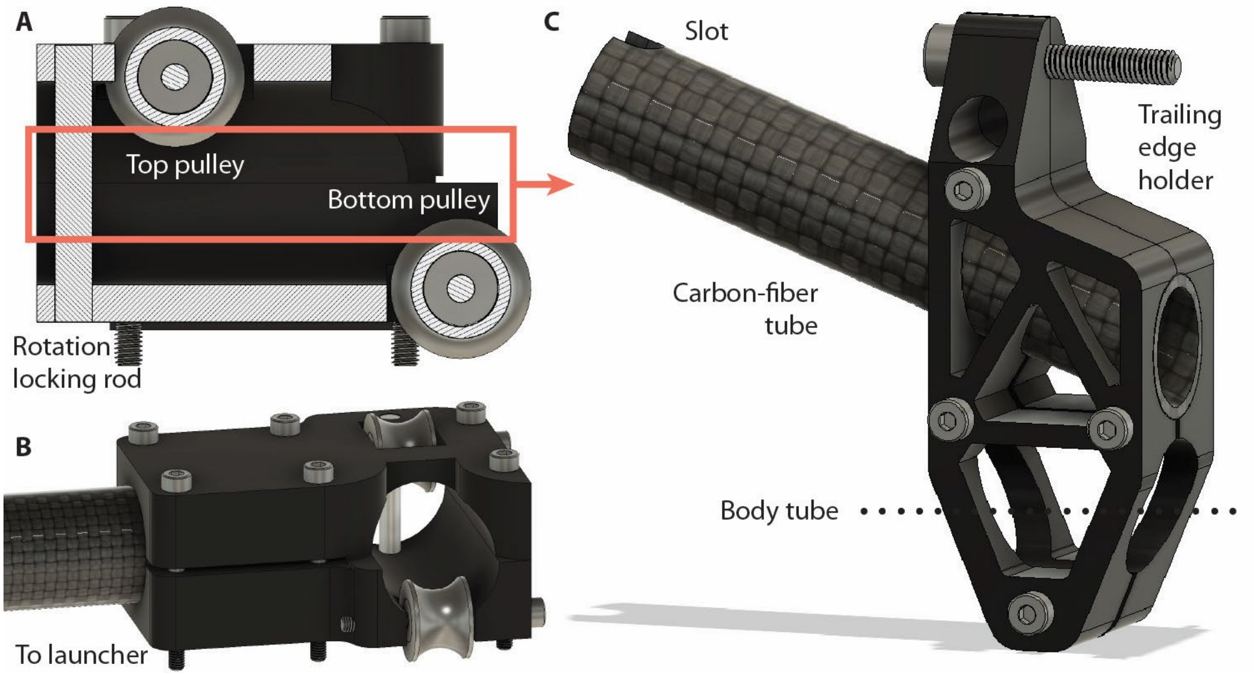

Supplementary Fig. 11. Launch adapter.

A. Section view of the launcher-bird interface mechanism. The robot features a slotted carbon fiber tube that slides in-between the top and bottom pulleys. The horizontal offset of the pulleys of 30 mm is sufficient to support the clockwise torque imposed by the robot's weight. **B**. 3D view of the launcher connection on the robot. Weight minimization is more critical as this sub-assembly counts towards the bird's payload, hence the lightened 3D printed bracket and 60 mm carbon fiber tube. The bracket also holds the trailing edge of the wing, serving as a double function system. The launcher connection is therefore placed as far forward as possible to minimize torque. Placement of the system any further forward would be impeded by the wing. The end of the carbon fiber tube is slotted to lock the rotation. **C**. 3D view of the launcher adapter, showing the double ball bearing assembly and the rotation lock rod at the bottom. The large carbon fiber tube goes leftwards towards the launcher, giving a 40 cm clearance, sufficient to launch without any risk of contact.

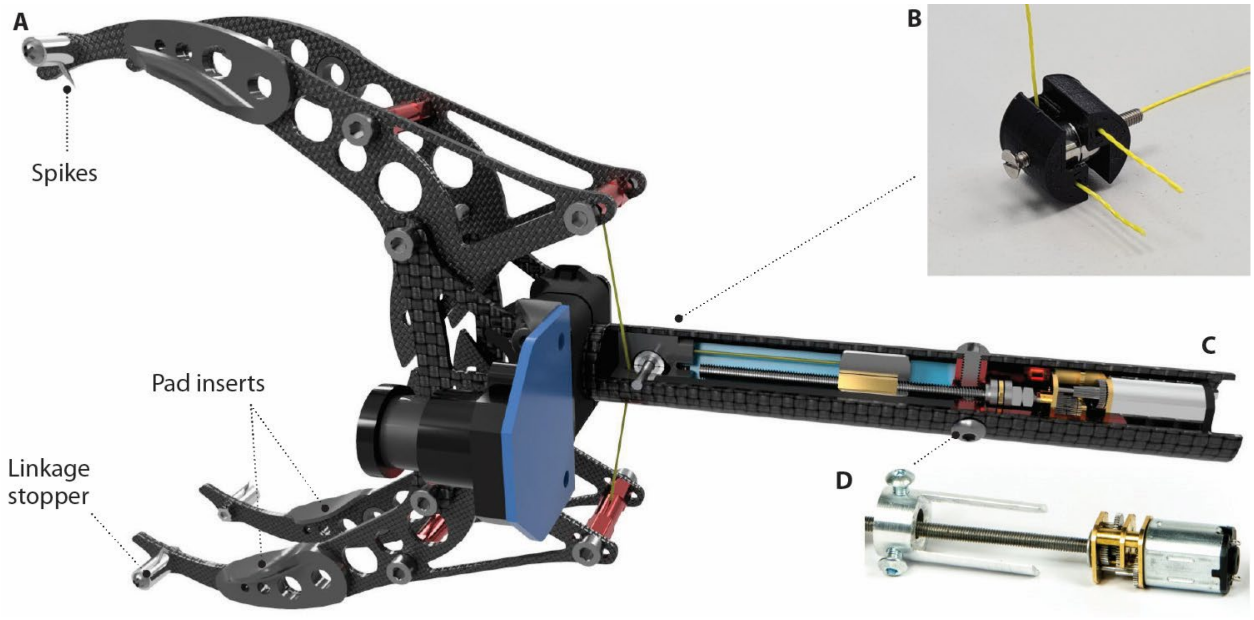

Supplementary Fig. 12. 3D view of the re-opening mechanism.
A. The eco-flex-spike system is composed of a silicone membrane that is fixed to four PLA inserts that are screwed to the carbon-fiber claws. These pad inserts offer a large surface area, both for contact with the branch and bonding of the membrane. The spikes are 1 mm steel pins fitted on standard metal linkage stoppers. The membrane presses against the spring (not represented here). **B**. View of the tendon diverter from the leg main axis towards the claw-back extremities. **C**. The 3D cut view of the re-opening mechanism located within the leg of the robot. **D**. View of the motor, reduction, and leadscrew with the anti-rotation aluminum holder which takes the load of the whole mechanism.

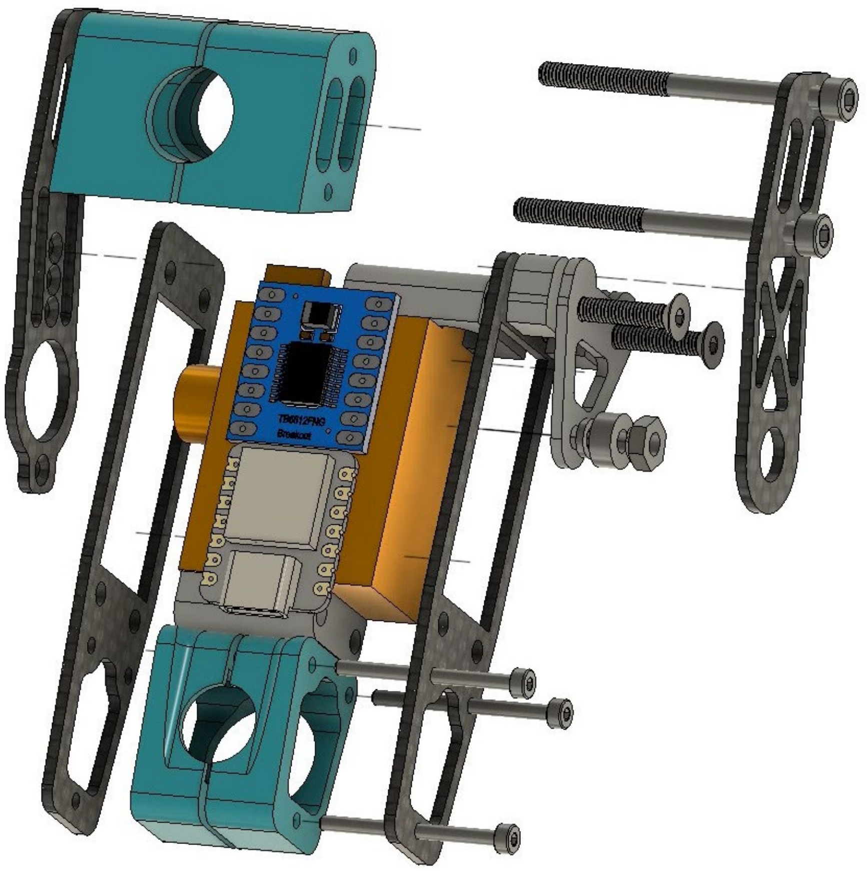

Supplementary Fig. 13. Leg exploded view.
3D view of the servo assembly which constitutes the hip joint. The left side attaches to the servo mount (hidden) while the right side is fixed to a ball-bearing pivot joint. Two compression parts (green) ensure fast and adjustable mounting both to the tail tube and the leg tube. The leg electronics are mounted onto the servo, between the carbon side plates.

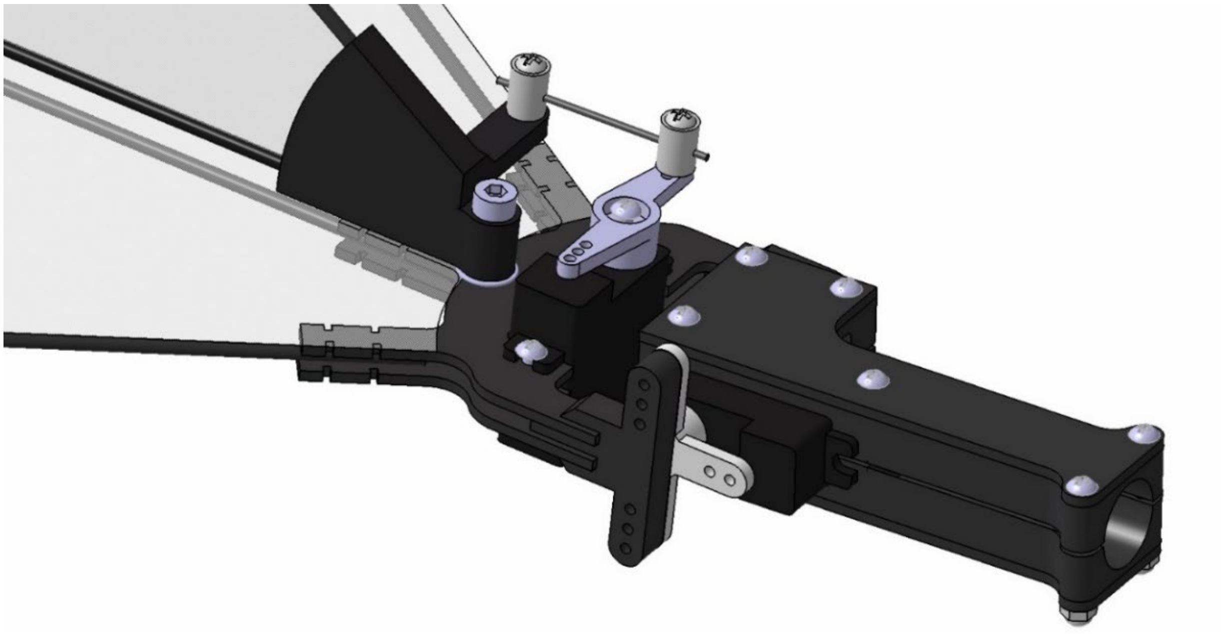

Supplementary Fig. 14. Tail assembly view.
3D-view of the tail assembly. The horizontal elevator is mounted on the elevator servo (black), on one side to the servo horn, and on the backside to a bearing back pivot (hidden). The tail (not shown) is fixed to the tail tube through compression of the 3 through-hole M2 screws. The vertical rudder pivot around a 3 mm shaft fixed in the elevator, parallel to the servo axis.

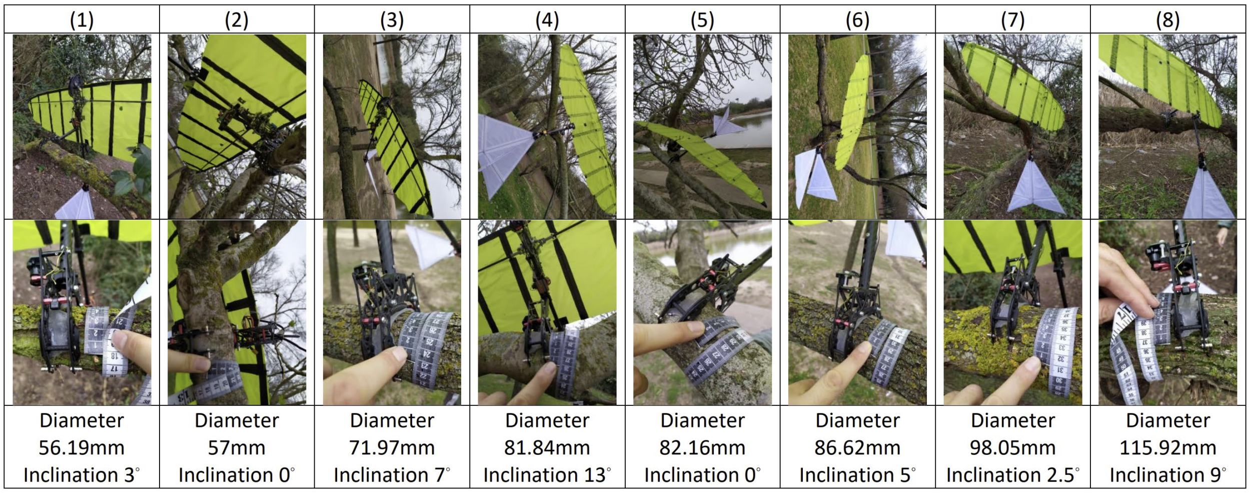

Supplementary Fig. 15. Outdoor branch grasping

The grasping of the claw on different branches; from subfigure (1) to (8), the branch size changes from 56.19mm to 115.92mm.

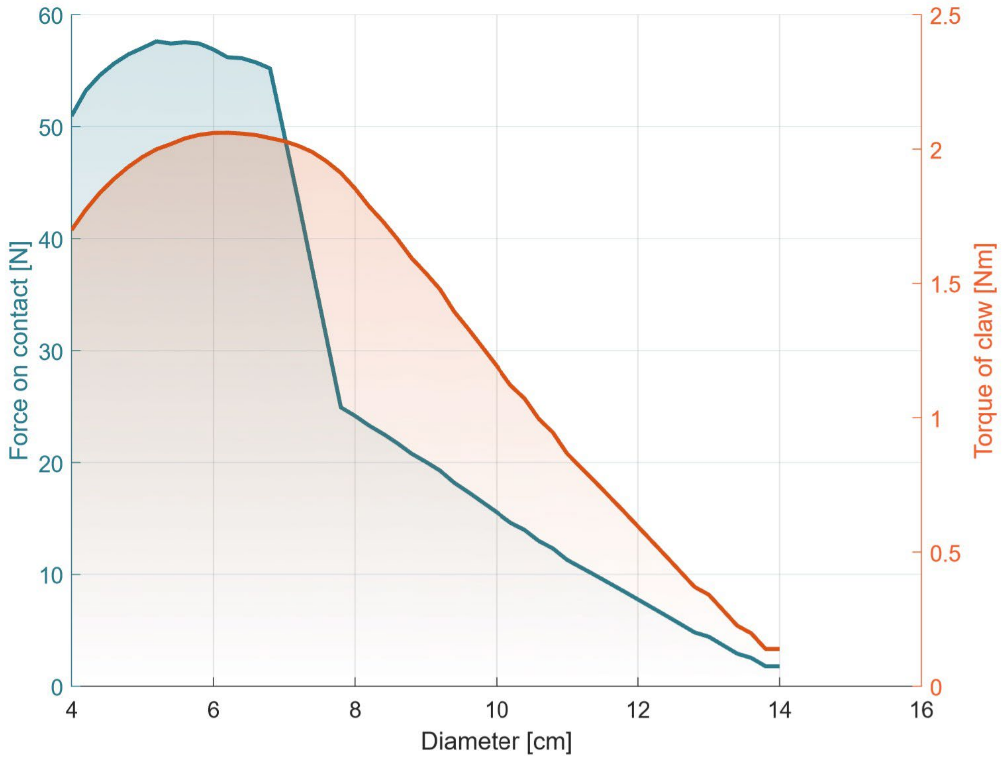

Supplementary Fig. 16. Branch diameter parametric analysis.

The red line is the torque of the claw around its rotation axis. Its maximum aligns with the branch diameter of 6 cm in use in the perching flight experiments. The blue line is the perpendicular force at the point of contact with the branch. It can be seen that there is a discontinuity at 7 cm when the point of contact swaps from the inner part of the claw to the spike. Up to a 12 cm diameter, the forces at the branch contact point remain higher than the robot's weight of 7 N.

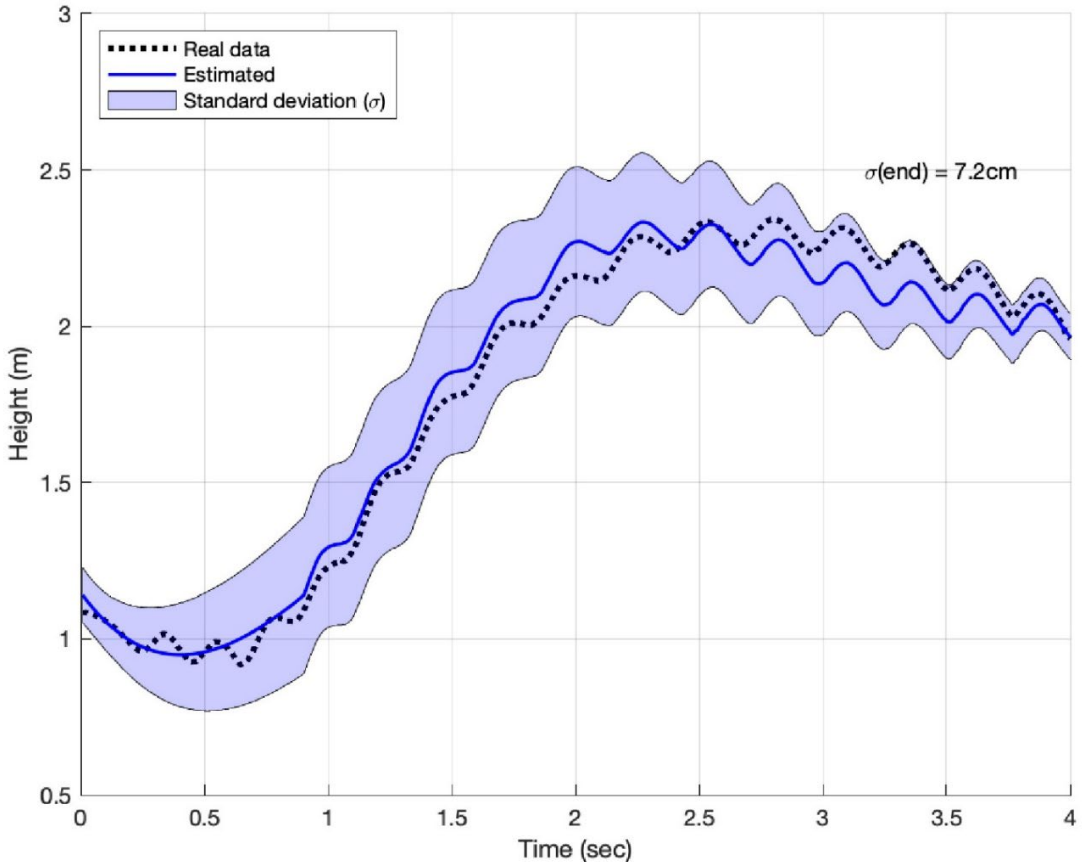

Supplementary Fig. 17. Model response vs real response in typical flight.
Model response vs real response in a typical branch experiment (see Supplementary Table 4). The filled zone indicates the statistical confidence of the sensitivity analysis, whose boundary is measured by the standard deviation (σ). The value σ(end) refers to the last time step.

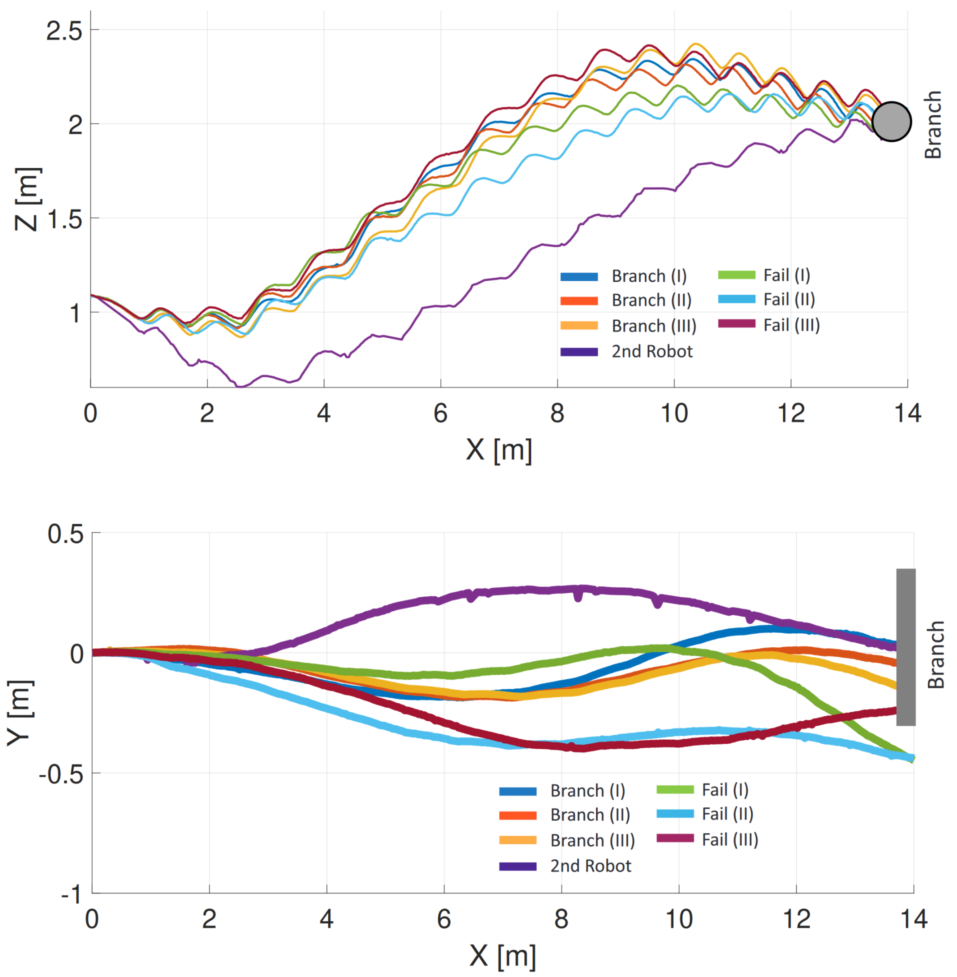

Supplementary Fig. 18. Failed flight trajectories

Trajectories in the vertical plane (top) and horizontal plane (bottom) of all the branch perching flights, including the 3 failed flights. Analysis of the plots shows that the first two failed flights missed the branch to its right. The third failed flight grazed the top of the branch, with a trajectory slightly too high.

Voltage regulation

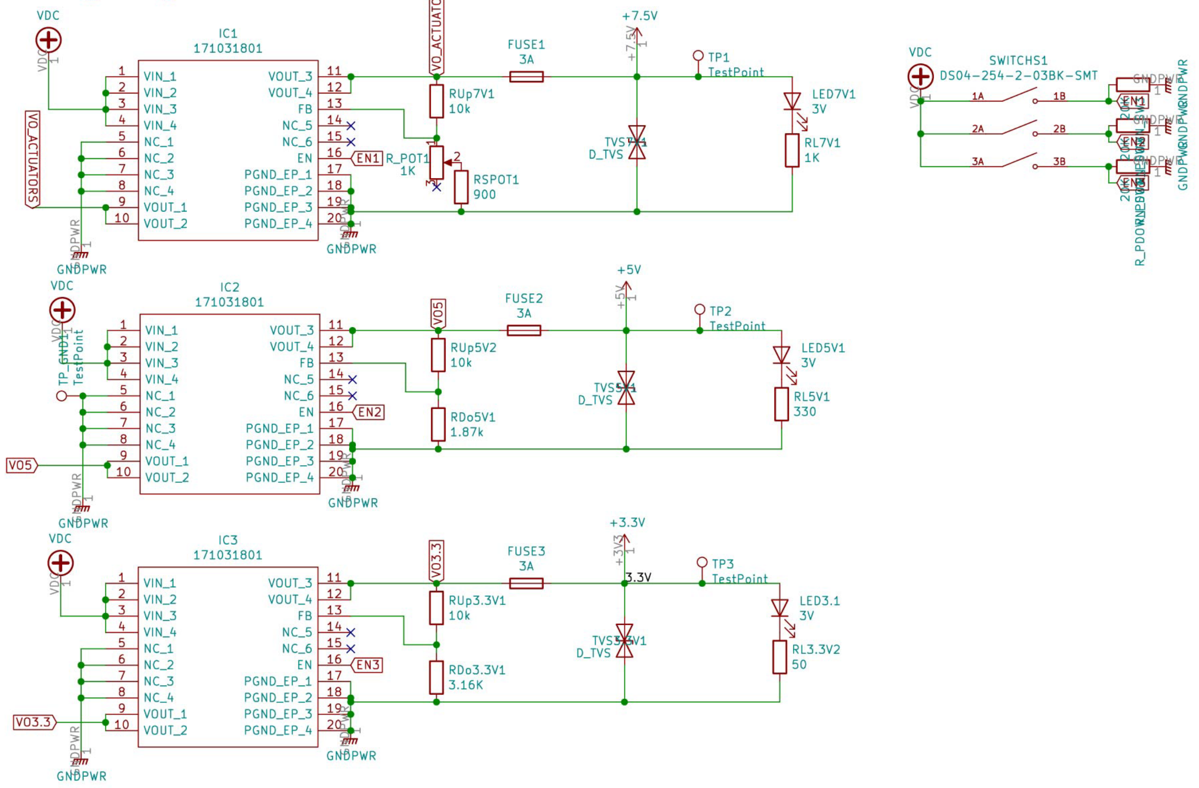

Motion tracking LEDs

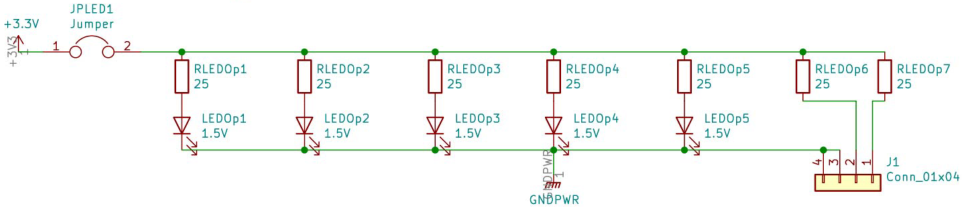

Connectors

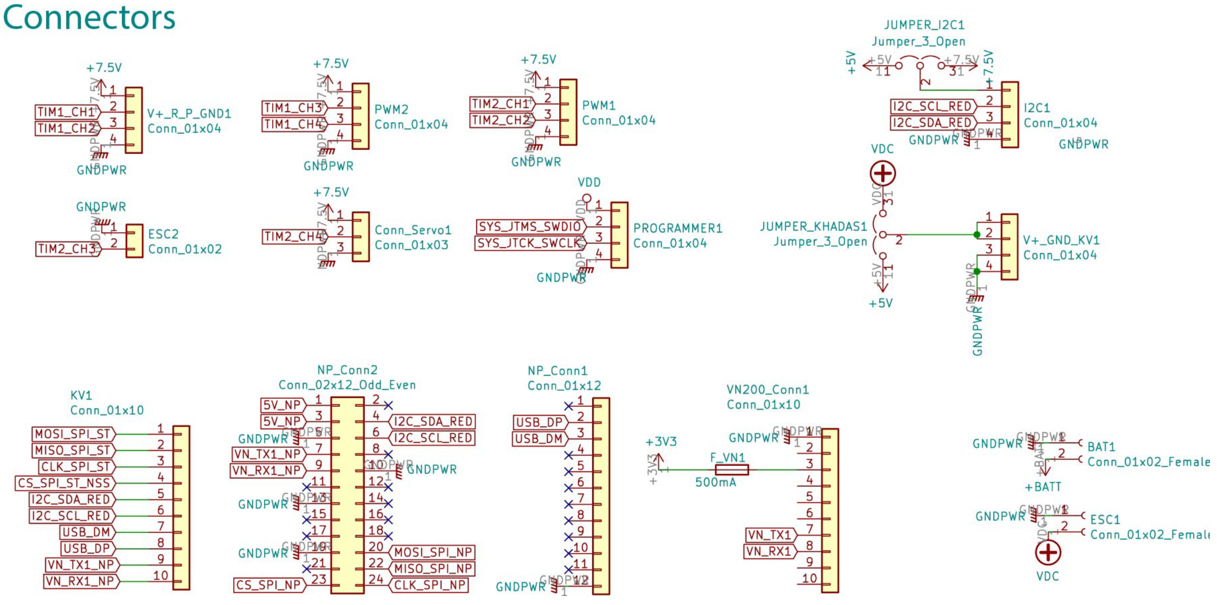

Microcontroller

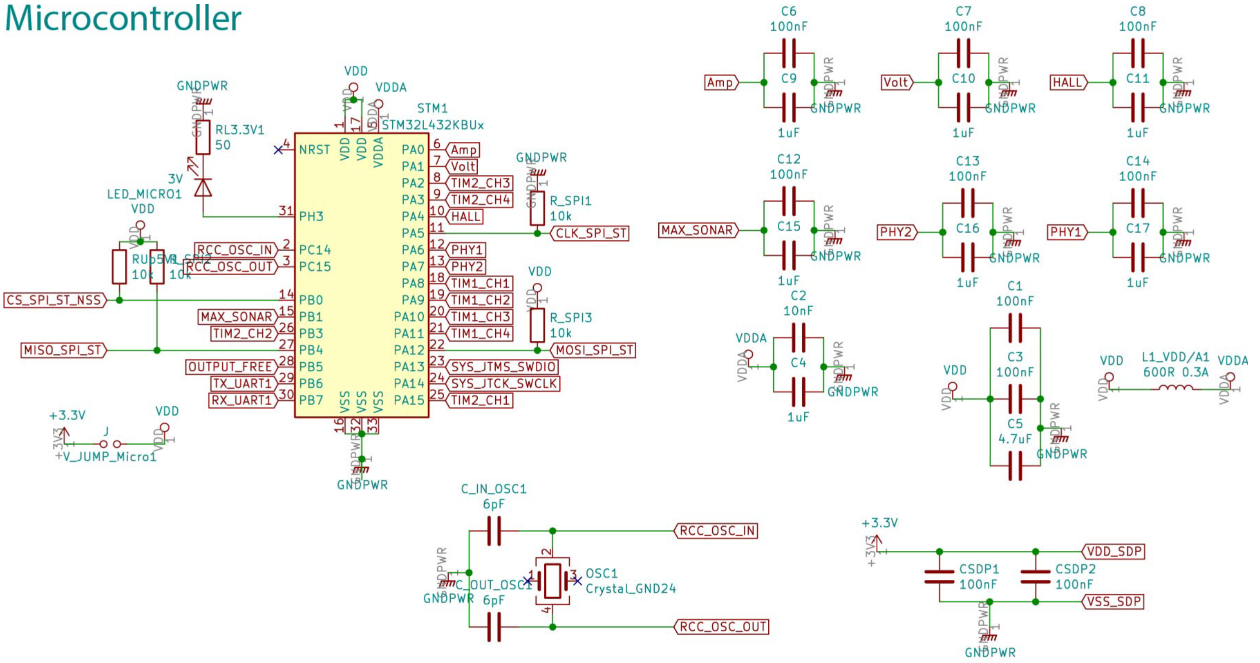

Sensors

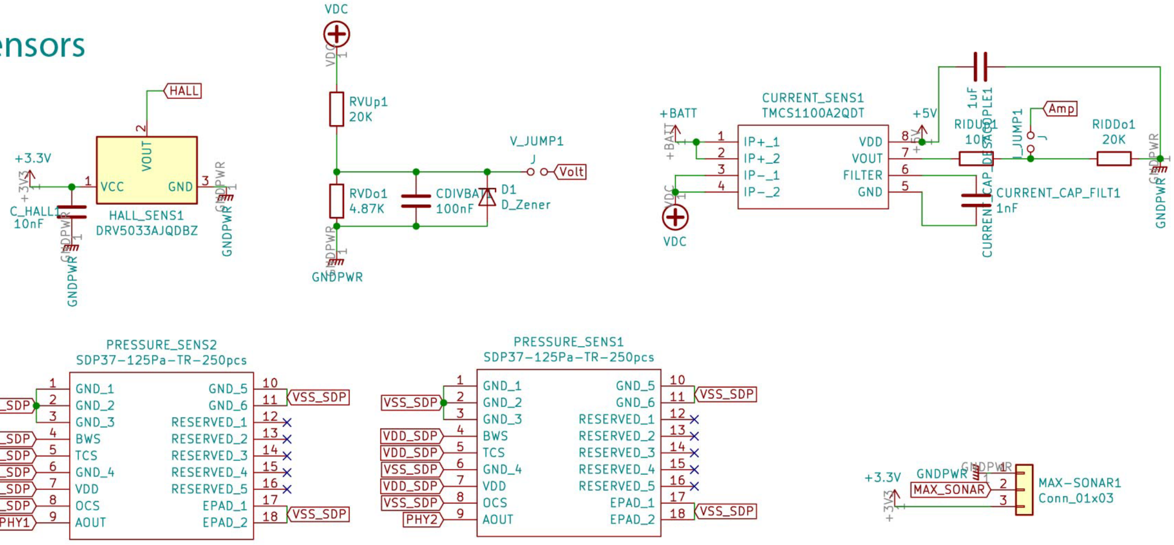

Communication

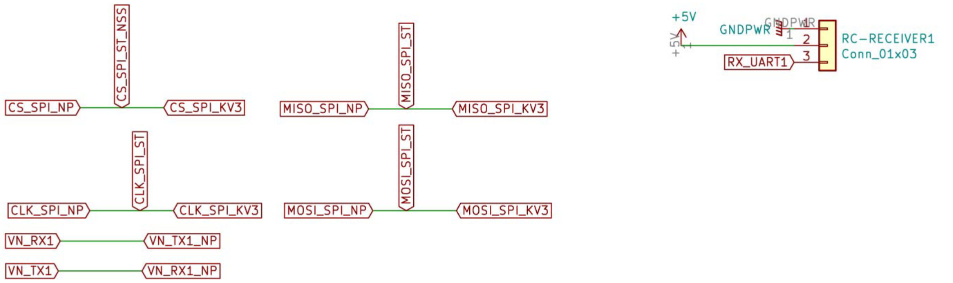

Supplementary Fig. 19. Electronics schematics.

Full electronics schematics of the electronics in the body of the flapping-wing robots. Please contact the authors for the corresponding PCB layout.
