# Supplementary material for: How ornithopters can perch autonomously on a branch
Source: Nat Commun. 2022 Dec 13;13:7713. doi: 10.1038/s41467-022-35356-5 (PMC9747916; doi:10.1038/s41467-022-35356-5)
Supplement: Supplementary file 3 — Description of Additional Supplementary Files [file 41467_2022_35356_MOESM3_ESM.pdf]

LIST OF ADDITIONAL SUPPLEMENTARY FILES

How ornithopters can perch autonomously on a branch

File name: Supplementary Movie 1
Description: High-speed claw closing.

File name: Supplementary Movie 2
Description: Claw reopening with current measure.

File name: Supplementary Movie 3
Description: Active leg oscillation experiment.

File name: Supplementary Movie 4
Description: Active leg launcher experiment.

File name: Supplementary Movie 5
Description: Claw impact launcher experiment.

File name: Supplementary Movie 6
Description: Soft branch perching flight experiment.

File name: Supplementary Movie 7
Description: Branch perching flight experiments.

File name: Supplementary Movie 8
Description: Slow-motion branch perching flight experiments.

File name: Supplementary Movie 9
Description: Motion capture view of perching flight.

File name: Supplementary Movie 10
Description: 2nd robot perching demonstration.
